# Supplementary material for: Patient Experiences of Treatment Deferral for Evaluation and Monitoring after a Trace Xpert Ultra Result
Source: medRxiv. 2025 Jan 23:2025.01.22.25320963. Preprint. [Version 1] doi: 10.1101/2025.01.22.25320963 (PMC12077874; doi:10.1101/2025.01.22.25320963)
Supplement: 1 [file NIHPP2025.01.22.25320963V1-supplement-1.pdf]

medRxiv preprint doi: https://doi.org/10.1101/2025.01.22.25320963; this version posted January 23, 2025. The copyright holder for this preprint (which was not certified by peer review) is the author/funder, who has granted medRxiv a license to display the preprint in perpetuity.
It is made available under a CC-BY 4.0 International license .

# SUPPLEMENTARY DATA:

Figure S1: *Ratings, by people with trace Ultra sputum results surveyed at the one-month follow-up visit, of the value and unpleasantness of each diagnostic test completed at baseline*

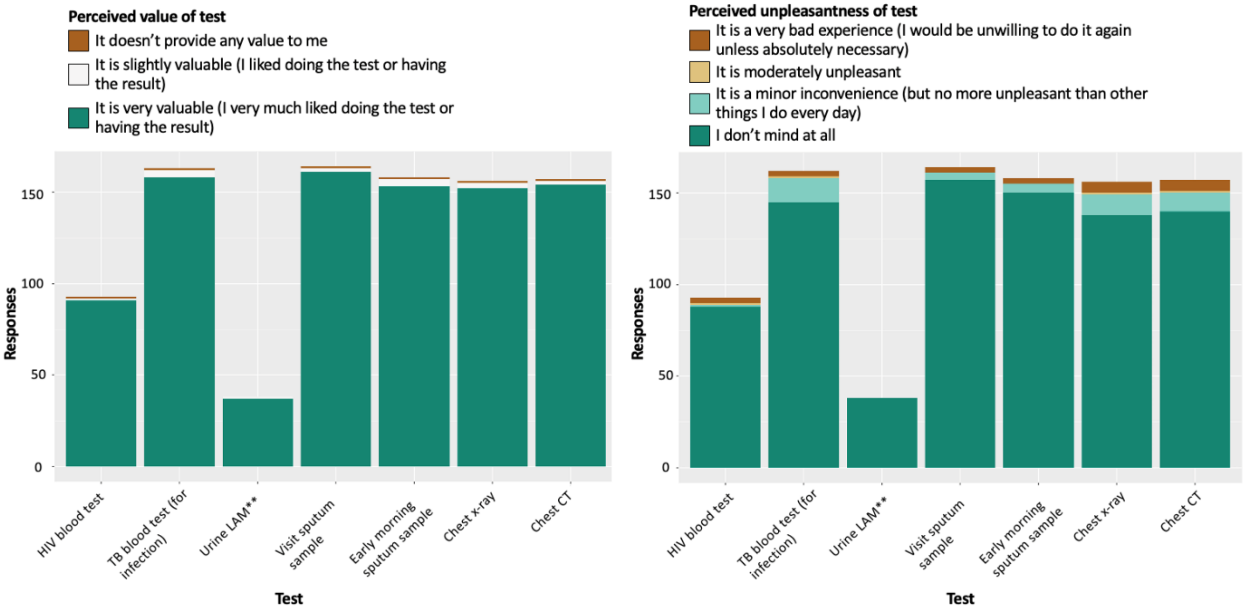


*For patients without known HIV only
**For patients with HIV only
Number of responses differ by test as ratings only included for participants who had completed that test during the baseline evaluation.
